# Liver Monocytes and Kupffer Cells Remain Transcriptionally Distinct during Chronic Viral Infection

**DOI:** 10.1371/journal.pone.0166094

**Published:** 2016-11-03

**Authors:** Martijn D. B. van de Garde, Dowty Movita, Marieke van der Heide, Florence Herschke, Sandra De Jonghe, Lucio Gama, Andre Boonstra, Thomas Vanwolleghem

**Affiliations:** 1 Department of Gastroenterology and Hepatology Erasmus University Medical Center, Rotterdam, The Netherlands; 2 Janssen-Pharmaceutica NV, Beerse, Belgium; 3 Department of Molecular and Comparative Pathobiology, The Johns Hopkins University School of Medicine, Baltimore, Maryland, United States of America; Ospedale San Raffaele, ITALY

## Abstract

Due to the scarcity of immunocompetent animal models for chronic viral hepatitis, little is known about the role of the innate intrahepatic immune system during viral replication in the liver. These insights are however fundamental for the understanding of the inappropriate adaptive immune responses during the chronic phase of the infection. We apply the Lymphocytic Choriomenigitis Virus (LCMV) clone 13 mouse model to examine chronic virus-host interactions of Kupffer cells (KC) and infiltrating monocytes (IM) in an infected liver. LCMV infection induced overt clinical hepatitis, with rise in ALT and serum cytokines, and increased intrahepatic F4/80 expression. Despite ongoing viral replication, whole liver transcriptome showed baseline expression levels of inflammatory cytokines, interferons, and interferon induced genes during the chronic infection phase. Transcriptome analyses of sorted KC and IMs using NanoString technology revealed two unique phenotypes with only minimal overlap. At the chronic viral infection phase, KC showed no increased transcription of activation markers *Cd80* and *Cd86*, but an increased expression of genes related to antigen presentation, whereas monocytes were more activated and expressed higher levels of *Tnf* transcripts. Although both KCs and intrahepatic IM share the surface markers F4/80 and CD11b, their transcriptomes point towards distinctive roles during virus-induced chronic hepatitis.

## Introduction

Detailed knowledge of intrahepatic immune responses is crucial for a better understanding of the processes underlying immunopathology. Chronic viral hepatitis induced by the Hepatitis B (HBV) and hepatitis C (HCV) virus affects almost 500 million people worldwide and leads to progressive liver fibrosis, decompensated cirrhosis, and hepatocellular carcinoma [1]. Due to ethical constraints, studies of liver residing leukocytes are seldom performed in patients, although these cells are essential in determining the outcome of the infection. As alternative, the Lymphocytic Choriomeningitis Virus (LCMV) mouse model can be used. LCMV clone (Cl) 13 infection in mice is an established small animal model for immunological studies on persistent viral infection such as HIV, but also HBV and HCV [2]. The ability of LCMV to infect hepatocytes, among other cells, underlines the relevance of this model for the study of virus induced hepatitis [3–7].

The largest innate immune cell population in the liver are the tissue-resident macrophages, also known as Kupffer cells (KC). KCs are abundantly present in the liver sinusoids, are crucial players in maintaining tissue homeostasis, and form together with sinusoidal endothelial cells the first barrier for pathogens to enter the liver [8]. KCs can respond to danger signals using a variety of pathogen recognition receptors, such as Toll like, scavenger and antibody-receptors and, depending on the local environment, initiate an inflammatory response, or induce tolerogenic T-cell responses [9, 10]. Previously, we described that liver inflammatory monocytes resembled KC but were functionally distinct after 24 hours of LCMV Cl13 infection in mice. Both cell types showed an activated phenotype with increased transcription of activation markers *Cd80* and *Cd86*, and inflammatory cytokines *Tnf* and *Il6* [6].

Monocytes patrol the body for inflammatory foci and are therefore among the first cells to respond to inflammation. They are quickly recruited in great numbers thereby shaping the immune environment [6, 11]. These early monocytes are recruited in a CCL2/CCR2-dependent manner and are phenotyped in mice as F4/80^+^CD11b^+^CCR2^hi^Ly6C^hi^CX3CR1^low^. They can exert pro-inflammatory and antimicrobial functions, such as secretion of inflammatory cytokines IL-6 and TNF [6, 12, 13]. Previously, we showed that e*x vivo* HBsAg stimulation of blood monocytes revealed high cytokine induction [14]. However, chronic HBV patient derived blood monocytes were not activated despite abundant viral proteins in their plasma [14]. The role of liver monocytes and possible regulatory mechanisms controlling monocyte activation during chronic infection are still elusive.

KCs and monocytes are cells with high plasticity and can exert diverse functions depending on their environment. In mice, KCs have been shown to induce tolerogenic T-cells after phagocytosis of particle-bound antigens under homeostatic conditions, whereas monocytes showed no or minimal particle uptake. However, during early inflammatory conditions, monocytes were fit to counteract the tolerogenic KCs by taking up particles and producing TNF and inducible nitric oxide synthase [10]. Experiments on *Listeria monocytogenes* infection revealed that monocytes replenish dead KCs in the liver and exert an inflammatory response, followed by a tissue-repair response to restore homeostasis [15]. On the other hand, monocytes have also been described as regulatory cells that can suppress CD8+ T cell proliferation during LCMV Cl13 infection in mice [16].

We previously showed that CD14^+^ cells derived from chronic HBV patient livers displayed an activated phenotype and are able to interact directly with hepatitis B surface antigen (HBsAg) [17]. *In vitro*, these CD14^+^ cells produce high amounts of tumor necrosis factor (TNF), interleukin (IL)-6, and CXCL8 after stimulation with HBsAg and can activate NK cells [17]. As both IM and KC are CD14 positive, the precise roles of both cells during a chronic infection still need further elucidation. In the current paper we set out to fully characterize the immunology related gene transcription of sorted KCs and IM during chronic LCMV infection as a surrogate model for chronic viral hepatitis. We investigate whether these cells are distinct populations at the transcriptome level, and examine the role and activation status of both cells throughout chronic infection.

## Materials and Methods

### Study design, mice and virus

LCMV Cl13 was obtained as a kind gift from E. Zuniga, University of California in San Diego. LCMV Cl13 was propagated in BHK21 cells and the titer was determined by plaque assay as previously described [18, 19]. Female C57BL/6 mice aged 4–6 weeks (Charles River, France) received 2 x 10^6^ plaque forming units (PFU) LCMV Cl13 intravenously (*i*.*v*.). Mice were co-housed with a maximum of 4 mice per cage and were fed *ad libitum*. Animals were maintained in a Biosafety level-III isolator according to Dutch national biosafety guidelines. Body weight and the assessment of clinical symptoms were determined 2–3 times a week (Table 1). Blood was drawn from 6 mice at indicated time points to assess serum cytokines and liver enzymes. Mice were sacrificed in groups of 4–6 at indicated time point for whole liver qPCR, Immunohistochemistry, FACS analyses, and cell sorting (S1 Fig). The study was approved by the animal ethics committee of the Erasmus University Rotterdam, and conducted according to relevant Dutch national guidelines.

**Table 1:** Clinical Scoring of LCMV infected mice.

Clinical Score	Observation
CS 0	Normal behavior, active, no aberrant fur
CS 1	Pilo-erection AND/OR mild ruffled fur
CS 2	Mild hunched posture OR mild ruffled fur AND slightly less active
CS 3	Hunched posture AND ruffled fur AND less active
CS 4	Hunched posture AND ruffled fur AND inactive (very low or absent mobility)
CS 5	Death

### Isolation of total liver non-parenchymal cells

Liver was removed without perfusion, cut into small pieces, and treated with 30 μg/ml Liberase TM (Roche) and 20 μg/ml DNAse type I (Sigma) for 30 min. Parenchymal cells were removed by low speed centrifugation at 50 g for 3 min and erythrocytes were lysed with 0.8% NH_4_Cl. The remaining non-parenchymal cells were resuspended in culture medium consisting of RPMI-1640 (Lonza) supplemented with 10% FCS (Sigma), 10 mM HEPES (Lonza), 2 mM L-glutamine (Lonza), 1% penicillin/streptomycin (Lonza) and used for further analysis.

### Plaque Assay

Parts of infected livers were weighed, homogenized using ceramic beads, and centrifuged for 10 min at 450 g at 4°C. Supernatant was serially diluted in Dulbecco’s modified Eagles Medium (DMEM, Lonza) supplemented with 10% FCS before inoculation onto overnight grown 90% confluent VeroE6 cells in 6 well plates (Corning). After 1 hour incubation, each 10^2 to 10^7 serial diluted inocula was removed and the cell layers were covered with a solution of 0.5% SeaKem®Agarose (Lonza) in Minimal essential Medium Eagle (EMEM, Lonza) supplemented with 10% FCS. The agarose layer was removed after 5 days incubation at 37°C, 5% CO_2_, and cells were stained with a crystal violet solution in 2% formaldehyde (Merck). The number of plaques at each dilution was counted and averaged to obtain the viral concentration of the sample in PFU/ml. Each sample was corrected for liver weight input and is expressed as PFU per gram liver.

### Flow cytometry

Total liver non-parenchymal cells were stained with Aqua Dead Cell Stain from Invitrogen, and antibodies directed against CD45 eFluor450 (30-F11), F4/80 APC (BM8), CD11b PECy7 (M1/70) from eBioscience and Ly6C APCCy7 (HK1.4) from Biolegend (unless otherwise indicated), and fixed with 2% formaldehyde for 1 hour, after which cells were analyzed using a FACSCanto-II flow cytometer and FACSDiva software (BD Biosciences).

### RNA isolation of liver homogenates, generation of cDNA and real-time PCR

Liver was homogenized in RNAlater (Qiagen). RNA was extracted using Trizol (LifeTech) and a NucleoSpin RNAII kit (Bioké). cDNA was generated using the iScript cDNA Synthesis Kit (Bio-Rad Laboratories) according to the manufacturer’s protocol. Quantitative PCR were performed using SYBR-green and MyIQ5 detection system (Bio-rad Laboratories). Sequences of primers are listed in Table 2. Expression of target genes was normalized to the expression of GAPDH using the formula 2^-ΔCt^, ΔCt = Ct_RNAX_-Ct_GADPH_.

**Table 2:** Gene-specific primers used for whole liver RNA analyses.

**Taqman gene expression primers**			
**Gene ID**	Primer ID*		
*Ifng*	Mm01168134_m1		
*Gapdh*	Mm99999915_g1		
**SYBR green primers**			
**Gene ID**	**NCBI ID**	**Direction**	**Primer sequence 5’– 3’**
*Il6*	NM_031168.1	F	TGGTGACAACCACGGCCTTCC
		R	AGCCTCCTGACTTGTGAAGTGGT
*Tnf*	NM_013693.2	F	CAGGCGGTGCCTATGTCTC
		R	CGATGACCCCGAAGTTCAGTAG
*Ifnb*	NM_010510.1	F	GCCTGGATGGTGGTCCGAGC
		R	ACTACCAGTCCCAGAGTCCGCC
*Isg15*	NM_015783.3	F	CAGGACGGTCTTACCCTTTCC
		R	AGGCTCGCTGCAGTTCTGTAC
*Oas12*	NM_011854.2	F	GGATGCCTGGGAGAGAATCG
		R	TCGCCTGCTCTTCGAAACTG
*Gapdh*	NM_008084,2	F	CGTCCCGTAGACAAAATGGT
		R	TCTCCATGGTGGTGAAGACA

*Primer/probe mixes from Life-technologies

### RNA isolation of sorted cells and NanoString

KC and IM from 4–6 pooled livers were purified at day 0 (in duplicate), day 15, day 21, and day 41 post infection (p.i.), by cell sorting based on the expression of F4/80, CD11b and Ly6C, after initial enrichment using CD45 PE followed by anti-PE Microbeads (Milteny Biotec) selection. Following staining, cells were fixed with 2% formaldehyde for 1 hour, and sorted on a FACS Aria SORP flow cytometer (BD Biosciences). Total RNA was isolated from sorted cells using the RNeasy FFPE kit (Qiagen) following manufacturers’ protocols starting with adding 150 μl Buffer PKD, to reverse any possible formaldehyde induced RNA modification. The nCounter GX Mouse Immunology Kit (NanoString Technologies, Seattle, WA, USA) was used to measure the expression of 561 genes in the RNA samples. Following hybridization, transcripts were quantitated using the nCounter Digital Analyzer. Samples were run by the Johns Hopkins Deep Sequencing & Microarray Core. To correct for background levels, the highest negative control value for each sample was subtracted from each count value of that sample. Following background subtraction, any negative count values were considered as 0. Values were normalized by the geometric mean of 13 housekeeping genes provided by the company panel.

### Immunohistochemistry for F4/80

Liver was fixed in 4% formaldehyde, embedded in paraffin, and cut into 5 μm sections. F4/80 antigen was retrieved using Proteinase K (Sigma). Endogenous peroxidase was inactivated using 3% hydrogen peroxide (Dako). Liver sections were incubated with rat anti-F4/80 antibody (eBioscience) and rabbit anti-rat HRP (Dako). Upon addition of DAB, liver sections were counterstained with hematoxyline (Merck).

### Cytokine measurement

Serum TNF, IL-10, IFNγ and CXCL1 levels were measured using the MSD® MULTI-SPOT Assay System, Mouse Pro-Inflammatory 7-plex Ultra-Sensitive Kit (MesoScaleDiscovery, ref. K15012C-2), following the instruction manual. After an overnight incubation at 4°C, samples were measured in monoplicate undiluted and standard curve and blanks in duplicate. Electrochemiluminescence was read on a Sector Imager 6000 (MesoScaleDiscovery).

### ALT measurement

Serum ALT levels were measured using an ELISA kit for Alanine Aminotransferase (Biotang) according to the manufacturer’s protocol.

### Data analysis and statistics

Differences between groups were calculated using one-way ANOVA with Dunn’s Multiple Comparison post-test (GraphPad Prism version 5.01; GraphPad Software). Differences were considered significant when P < 0.05. Results are presented as the mean ± SEM, unless otherwise indicated. Principal component analyses was performed on whole log 2 transformed data set using Multi-experiment viewer (MeV) software version 4.9 and hierarchical clustering was executed using one minus pearson correlation in GENE-E software version 3.0.204 (Broad Institute, Inc). The statistical variance of all transcripts of 2 KC samples at day 0 were determined. The fold change of these values was calculated and used as cutoff to determine differentially expressed genes (1.27-fold for KC). The cutoff for differentially expressed genes in IM was determined similarly (1.15-fold for IM). Non-expressed genes were defined as <100 relative RNA counts and below four times the standard deviation in all samples.

## Results

### LCMV clone 13 infection induces evident hepatitis accompanied by changes in F4/80 expressing cells

**Fig 1:**
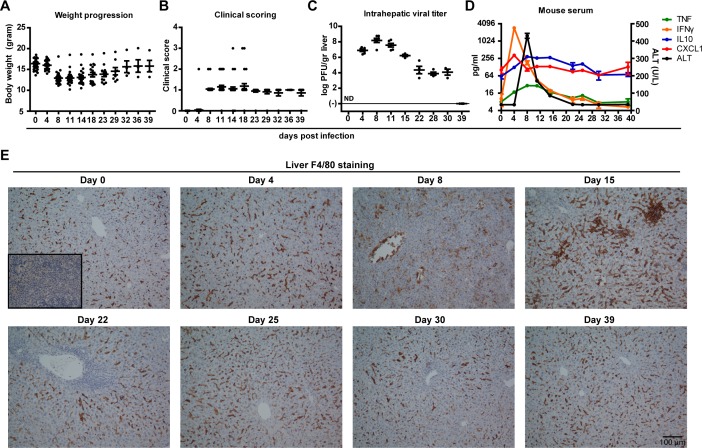
Evident LCMV-induced hepatitis with overt clinical symptoms, rise in serum cytokines and intensified F4/80+ cell staining. During the course of LCMV Cl13 infection mice were weighed (A) and clinically scored using predefined criteria (Table 1) (B). At regular intervals mice were sacrificed to determine the intrahepatic LCMV viral load (C). Serum was collected at different time points to measure ALT (D) and cytokines TNF, IFNγ, CXCL1, and IL10 (D) using a multiplex assay. X-axis shows days post infection (A-D). Error bars indicate mean ± SEM (A-D). ND, not determined (C). F4/80 IHC staining was performed at indicated time points to characterize the presence, morphology and localization of F4/80+ cells within the liver (E). Insert at day 0 shows non-primary control staining of mouse spleen (E). Scale bar indicates 100 μm (E).

Previously, we showed that LCMV Cl13 is able to infect the liver in addition to other organs [6]. Infection with LCMV results in active intrahepatic replication, which can last up to 40 days [20]. The progression of infection was accompanied by wasting (Fig 1A, p<0.001 at peak of infection) and increased clinical scores based on set criteria (Table 1, and Fig 1B, p<0.001 at peak of infection), which aggravated just after the peak of infection around day 14 p.i. and recovered towards viral clearance from the liver at day 39. At peak of infection, intrahepatic LCMV titers reached 8 log PFU/gr liver, which dropped to an average of 4 log PFU/gr liver from day 15 onwards (Fig 1C). LCMV-specific T cell exhaustion is at highest level and sustained from day 15 p.i., which is therefore regarded as the onset of chronic infection in this model [21]. Peak of intrahepatic LCMV replication was associated with evident rise of serum ALT, preceded by peak in IFNγ and CXCL1, and coincided with TNF and IL-10 levels (Fig 1D).

### No increased expression of cytokine and interferon in whole liver during LCMV-induced chronic hepatitis

**Fig 2:**
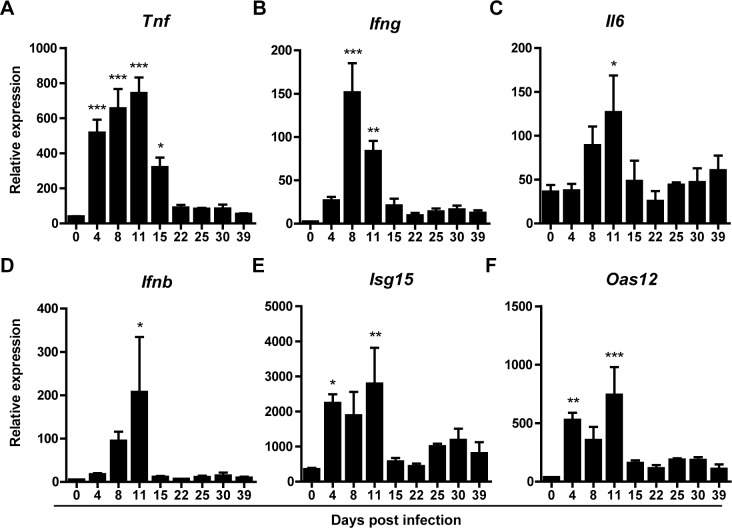
Hepatic cytokine and interferon transcript levels increase during the peak of LCMV replication but normalize thereafter. Whole liver RNA was isolated from LCMV infected mice at regular intervals and analyzed for transcription of inflammatory cytokines *Tnf*, (A) *Ifng* (B), *Il6* (C), *Ifnb* (D) and interferon induced genes *Isg15* (E) and *Oas12* (F) using qPCR. Given values on y-axes are relative expression to GAPDH. X-axes shows days post infection. Error bars indicate mean ±SEM. Significance of each time point was assessed using one-way Anova with Dunnett’s Multiple comparison test to day 0. *p<0.05, **p<0.01, ***p<0.001.

We next examined the impact of LCMV-induced hepatitis on liver-specific innate immune responses. Increased levels of pro-inflammatory cytokine transcripts occurred before (*Tnf*), during (*Ifng*), or after (*Il6*) the peak of intrahepatic viral replication (Fig 2A–2C). Interestingly, these levels were not significantly different from baseline during the chronic phase of the infection. A similar intrahepatic expression pattern was seen in transcript levels of interferon (IFN)-beta and selected IFN stimulated genes (ISG, *Isg15* and *Oas12*) (Fig 2D–2F). Despite ongoing LCMV replication from day 15 onwards, gene expression of ISGs, interferons, and pro-inflammatory cytokines were not significantly different from baseline levels.

### Kinetics of two distinct liver F4/80+ cell populations during chronic LCMV-induced hepatitis

**Fig 3:**
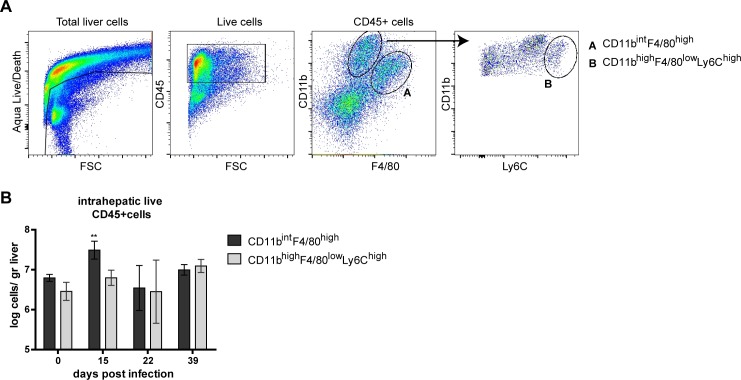
Kinetics of distinct F4/80+ cell populations from LCMV infected mouse livers. Mice were infected with LCMV Cl13 and were sacrificed at day 15, 22 or 39 p.i.. Distinct cell populations were determined using FACS analysis gating strategy of Live/CD45^+^F4/80^high^CD11b^int^ and Live/CD45^+^F4/80^low^CD11b^high^Ly6c^high^ (A). Arrow indicates follow-up gate for the selection of Live/CD45^+^F4/80^low^CD11b^high^Ly6c^high^ (A). Quantification of F4/80^high^CD11b^int^ (black bars) and F4/80^low^CD11b^high^Ly6c^high^ (gray bars) cells as log cells per gram liver (B) isolated from mouse livers (n = 4–6). Statistical significance was assessed using one-way Anova with Dunnett’s Multiple comparison test to day 0. **p<0.01

Because F4/80 expressing cells showed clear histological changes despite normalized whole liver transcription for selected inflammatory signals, we analyzed the longitudinal changes of two distinct F4/80+ myeloid cell populations (CD45^+^F4/80^high^CD11b^int^ and CD45^+^F4/80^low^CD11b^high^Ly6c^high^ cells) by flow cytometry (Fig 3A). Both cell subsets were observed at baseline and during different chronic phases of LCMV infection. Compared to non-infected mouse livers, CD45^+^F4/80^high^CD11b^int^ cells increased significantly during the early chronic infection phase. In contrast, CD45^+^F4/80^low^CD11b^high^Ly6c^high^ were not significantly altered during chronic LCMV infection (Fig 3B). The increase in CD45^+^F4/80^high^CD11b^int^ cells coincided with the more intense F4/80 staining in liver at day 15 p.i. (Fig 1E). Irrespective of the phase of chronic LCMV infection both cell populations maintained their characteristic surface phenotype.

### F4/80^+^ cell populations keep a distinct KC and IM phenotype throughout chronic infection

**Fig 4:**
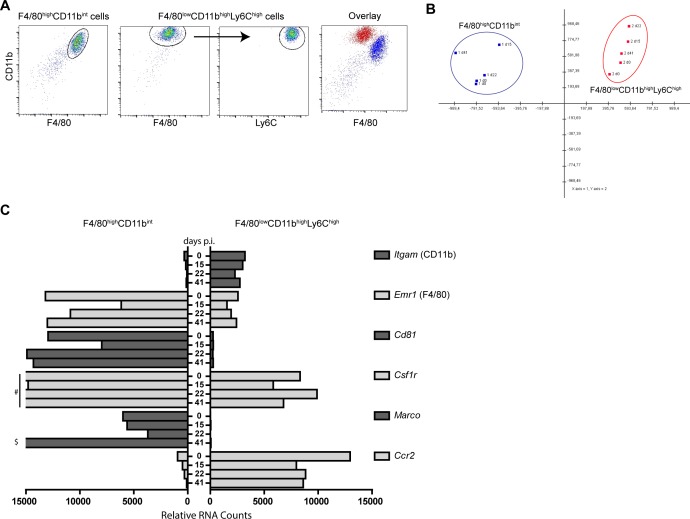
Transcriptomic changes during chronic LCMV-induced hepatitis in liver derived monocytes and Kupffer cells. Liver derived CD45^+^F4/80^+^CD11b^int^ KC and CD45^+^F4/80^int^CD11b^int^Ly6c^high^ IM were sorted and purity of sorted cells was assessed by FACS analyses and overlay graph (A). RNA was isolated from sorted cells at baseline (day 0) and after LCMV infection during the early chronic phase (day 15), chronic phase (day 22) and at time of viral clearance (day 41). Gene expression was measured using the nCounter GX Mouse Immunology Kit. Principal component 1 and 2 comprise 65% of the variance between samples (B). Transcription of myeloid cell defining markers for F4/80^+^CD11b^int^ (Left) and F4/80^int^CD11b^int^Ly6c^high^ (right) cells (C). Gene legend is indicated on the right side (C). Y-axis shows days post infection and X-axis indicates relative RNA counts (C). # 29297, 23963, 32090 relative RNA counts for *Csf1r* day 0, 22, and 41, respectively (C). $ 22720 relative RNA counts for *Marco* at day 41 (C).

We previously described distinctive functions for KC (phagocytic) and IM (TNF production) [6]. Plasticity is a well-known property of cells derived from the monocyte-macrophage lineage. Therefore we examined transcriptome profiles of F4/80^high^CD11b^int^ and F4/80^low^CD11b^high^Ly6c^high^ cells longitudinally. Cells were sorted from uninfected (day 0) and LCMV-infected mouse livers at the early chronic infection phase (day 15), during the chronic phase (day 22), and after clearance of LCMV from the liver (day 41). FACS analysis demonstrated highly pure non-overlapping cell populations (Fig 4A, and S1 Fig). Using the nCounter NanoString platform, transcripts of 547 immunology-related genes and 14 housekeeping genes were measured in sorted cells. Principal component analyses showed that 65% of the variance in gene expression could be explained using components 1 and 2, showing that both cells remained distinct on a transcriptome level throughout and after intrahepatic LCMV replication (Fig 4B). The surface expression of the discriminating markers used for FACS analyses of both populations, as shown in Fig 3, were reflected in the cell’s transcriptome. F4/80^high^CD11b^int^ transcribed high amounts of *Emr1* (F4/80) and low *Itgam* (CD11b) and vice versa for the F4/80^low^CD11b^high^Ly6c^high^ cells (Fig 4C and S1 Table). Furthermore, the F4/80^high^CD11b^int^ cells showed higher expression of macrophage markers such as *Marco*, *Cd81*, and *Csf1r*. The F4/80^low^CD11b^high^Ly6c^high^ cells expressed more *Itgam* and *Ccr2* at all time points (Fig 4C and S1 Table). These data unequivocally demonstrate that both F4/80+ cell populations remain distinctive during ongoing intrahepatic LCMV replication and confirm the KC and IM phenotypes of sorted cell populations.

### Kupffer cells and Infiltrating monocytes display a distinct viral antigen associated gene regulation

**Fig 5:**
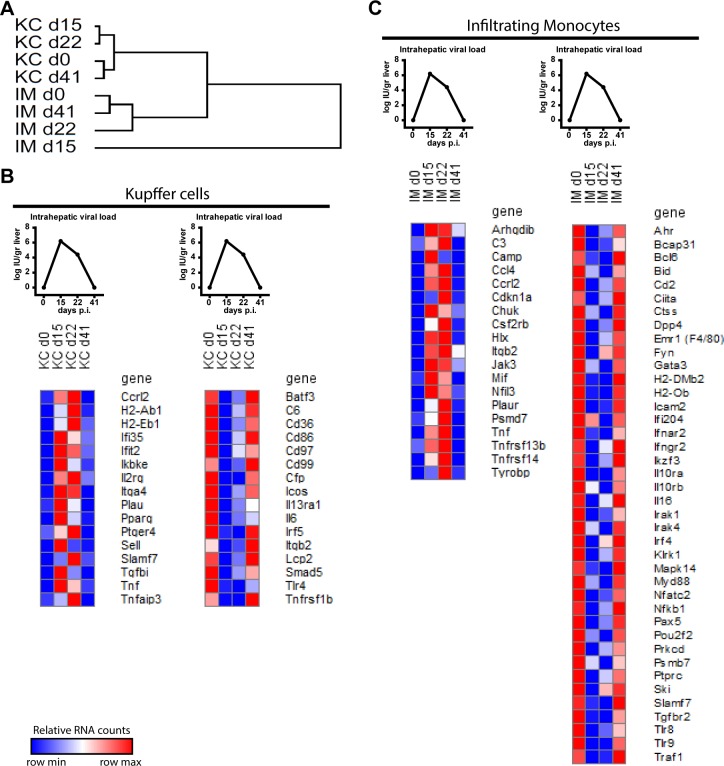
Kupffer cells and infiltrating monocytes exhibit a distinctive viral antigen associated gene expression. Hierarchical cluster of complete transcriptome of sorted KC and IM (A). Gene upregulation (left) and down regulation (right) associated with presence of viral antigens in the liver of KC (B) and IM (C). Viral antigen plots show simplified Fig 1C (B, C). Dark red indicates row max value, dark blue indicates row min value (B, C).

Hierarchial clustering of sorted KC and IM from different time points showed that the transcriptomes at baseline and viral clearance cluster together for both cell types (Fig 5A). This indicates that ongoing viral replication determines gene expression in both cells independently. We examined gene expression for up- and down-regulated patterns identical to the viral load pattern. Only 2 genes, *Ccrl2* and *Tnf*, showed a similar expression pattern in both cells (Fig 5 and S2 Fig). Among other genes, KC upregulated the ISGs *Ifi35* and *Ifit2*, and antigen presentation related genes *H2-Ab1* and *H2-Eb1*, which positively correlated with the presence of virus in the liver (Fig 5B). Inflammatory cytokine *Il6* and activation marker *Cd86* expression in KC negatively correlated to the presence of viral antigens in the liver (Fig 5B). IM showed among others increased *Ccl4* expression and decreased expression of *Tlr8* and *Tlr9* together with decreased inflammatory pathway molecules such as *Myd88*, *Nfkb1*, *Irak1*, *Irak4*. Furthermore, IM showed less transcription of several surface receptors including *Ifnar2*, *Ifngr2*, *Il10ra*, and *Il10rb* in the presence of viral antigens (Fig 5C). These data indicates that each cell type distinctively respond to the presence of viral antigens during chronic infection.

### IM and KC exhibit a distinctive activated transcriptional profile

**Fig 6:**
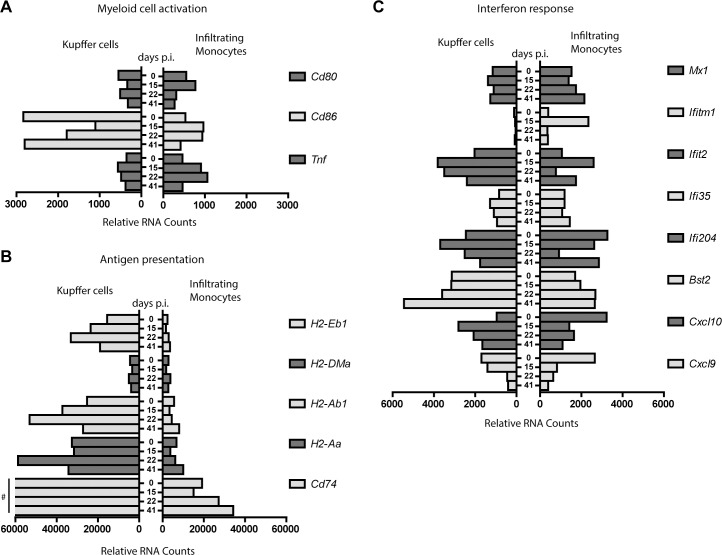
Kupffer cells and infiltrating monocytes display a distinct activation and functional transcriptomic profile. Gene expression profile of KC (left) and IM (right) related to myeloid cell activation (A), antigen presentation (B), and interferon response (C). Gene legend is indicated on the right side of each panel. Y-axis shows days post infection and X-axis indicates relative RNA counts. # 75413, 106998, 171430, 113981 relative RNA counts for *Cd74* at day 0, 15, 22 and 41, respectively (B).

The activation status of myeloid cells is often assessed by the expression of costimulatory molecules CD80 and CD86, and the production of cytokines and chemokines [8]. Compared to IM, KC were less activated as indicated by a non-regulated expression of *Cd80* and decreased expression of *Cd86*, while an increased expression of *Cd80* at the early chronic phase and *Cd86* during the whole chronic phases pointed towards more activated IM (Fig 6A). Nevertheless, both cells transcribed more *Tnf* during chronic infection, albeit almost 2-fold higher in IM compared to KC (Fig 6A). Overall, these data point to a differential activation status of KC and IM despite extraction from the same tissue and viral antigen rich environment.

### KC, but not IM show a transcriptional specialization towards antigen presentation during chronic infection

Macrophages are able to present antigens [8]. Here we observed that KC strongly upregulated several genes associated with antigen presentation with highest expression during the chronic phase at day 22 p.i.. In comparison, IM showed an overall much lower expression of most antigen presentation-related genes (Fig 6B), indicating transcriptional differences between KC and IM and underlining the potential of KC to present antigens during chronic infection. IM showed exclusive expression (*Camp*, *Trem1*, *IL1r2*, *Ltb4r1*, and *Mbp)* and relative higher expression (*Plaur*, *Spn*, *Tgfbi*, *S100a8*, *S100a9*, and *Sell)* of a diverse set of immune-related genes, which comprised cytokines, membrane proteins and receptors, signal transduction molecules, compared to KC at baseline and during infection (S1 Table). However, this set of genes does not point to a specific pro- or anti-inflammatory signature for the sorted IM throughout infection.

### KC and IM show an ambiguous antiviral response during chronic infection

During chronic infection KC showed an increased expression of *Cxcl10*, another marker for immune activation, whereas IM downregulated their *Cxcl10* transcription (Fig 6C). *Cxcl10* is upregulated in response to interferons, however *Ifna*, *Ifnb*, and *Ifng* transcription was very low or absent in KC and IM at any time point during infection (S1 Table). Despite clear induction of *Cxcl10* in KC and reduction in IM, other ISGs showed an irregular regulation (Fig 6C), indicating an ambiguous antiviral response by KC and IM during chronic hepatitis.

## Discussion

Our current understanding of liver specific immune cells during virus-induced liver disease has been hampered by the limited possibilities studying patient derived liver samples and the lack of suitable animal models. Here we use a surrogate model for human viral hepatitis, based on the infection of mice with LCMV Cl13. We show that LCMV Cl13 induced overt clinical hepatitis, with rise in serum cytokines during acute but not throughout the chronic phase. Similarly, acute HBV patients present high TNF and IFNγ in serum, whereas chronic HBV patients show normal levels of these serum cytokines [22]. The model was used to study liver resident and infiltrating myeloid cells during chronic viral hepatitis.

In order to sort cells from LCMV infected mice in a BSL-1 environment, cells required formaldehyde fixation, inherently leading to RNA strand breaks. Extracted RNA yield allowed only a limited number of qPCR reads, related to their fragmentation. For these materials, Nanostring hybridization has however been shown to yield reproducible gene expression data [23]. To obtain sufficient RNA for gene expression analysis, livers were therefore pooled from 4–6 mice prior to cell isolation, staining, and flowcytometric sorting. Gene expression analysis of sample pools pick up large differences and flatten out biological variation of individual animals. Results are therefore more robust, have higher accuracy, but minor transcriptome changes might be missed [24].

We show by gene expression analyses of sorted KC and IM that these cells are distinct and maintain their respective phenotypes throughout the infection. Cell distinctive phenotypes were most evident by genes solely expressed (*Marco* and *Trem1*) by either cell type or genes regulated in opposite fashion (*Cxcl10* and *Cd86*) throughout infection, suggesting either distinct regulatory mechanisms or that either cell type complements the other to maintain a balanced overall immune response.

During chronic infection, KC showed a less-activated phenotype. Conversely, IM were more activated, as shown by the upregulation of *Cd80*, *Cd86*, and *Tnf*, albeit lower when compared to acute LCMV clone13 infection as previously shown [6]. Myeloid cells have been shown before to exert various functions ranging from maintaining homeostatic balance to initiating inflammation, regulating immune response, and tissue repair, depending on their milieu [25]. Here, we found that KC have an increased expression of transcripts related to antigen presentation, suggesting that these cells are better equipped to present antigens and activate T cells during chronic infection.

Previously, we showed that after 24 hours of LCMV infection, KC were capable of expressing *Ifng* and *Ifnb* [6]. Here we observed no transcription of IFNs in KC and IM during chronic infection, despite the local presence of viral antigens. However, KC showed increased transcription of *Cxcl10*. Increased CXCL10 levels in serum have been associated with immune activation and has been classically regarded as a marker of ISG responses in HCV infected hepatocytes [26–28]. Moreover, evident ISG response in KC and not in hepatocytes have been shown to predict treatment outcome in chronic HCV patients, suggesting that KC are crucial players during chronic hepatitis [29].

We previously showed that intrahepatic CD14^+^ cells (comprising both KC and IM) from chronic HBV patients display an activated phenotype, based on higher expression of CD40 and CD80 [17]. Moreover, these CD14^+^ cells had an increased *HLA-ABC* and *HLA-DR* transcription, which was also observed in KC isolated during chronic LCMV infection. In addition, upon encounter with HBV particles human liver non-parenchymal cells had an induced *IL1b*, *IL6*, *CXCL8*, and *TNF* transcription [30]. Here, highly pure KC derived from LCMV infected livers also showed increased *Tnf*, but decreased *Il1b* and *Il6* transcription. Furthermore, *Tnf* transcripts were much higher in sorted IM, indicating a balance of KC towards antigen presentation and less to pro-inflammatory cytokine production.

We showed that monocytes infiltrating the liver during LCMV-induced chronic hepatitis were activated and transcribed various genes including *Tnf* correlating with the presence of viral antigens. However, peripheral blood monocytes from chronic HBV patients remained unaffected by viral proteins and HBV DNA, although they can produce high amounts of IL-6 and TNF upon stimulation with HBsAg in vitro [14]. In HBV patients, anti-inflammatory IL-10 in serum has been shown to be elevated and decreases cytokine production by monocytes [14, 31, 32], suggesting that regulatory mechanisms are in play during chronic viral hepatitis. We found elevated serum IL-10 levels during chronic LCMV infection and an exclusive transcription of *Il10* in sorted KC and not IM suggesting a cell-specific regulation of immune responses (Fig 1D and S1 Table). Based on the gene expression data presented here no clear functional specialization can as yet be ascribed to intrahepatic IM during virus induced hepatitis. Their function might be tightly regulated between a pro- or non-inflammatory state. Further insight will require a selective depletion of either KC or IM during or before LCMV, without disturbing the entire mononuclear phagocyte system [33].

In summary, we show that LCMV Cl13 induces evident chronic viral hepatitis with limited intrahepatic cytokine and interferon responses during the chronic infection. LCMV-induced hepatitis is characterized by morphological changes and increased intensity of the F4/80+ cell population, designated as IM and KC. KC and IM are distinct cell populations before, during, and after chronic infection, with important differences in activation status, antigen presentation, and gene expression profile correlating with the presence of viral antigens. Overall, these data suggest that intrahepatic monocytes and KC play distinctive roles during chronic virus-induced hepatitis.

## Supporting Information

S1 Fig Schematic overview of experimental layout. For each experiment uninfected control mice were sacrificed at day 0 (A: n = 6, B: n = 6, C: n = 12). A total of 70 mice were i.v. challenged after clinical scoring and weighing with 2x10E6 PFU LCMV Clone 13, indicated by an arrow (A: n = 41, B: n = 15, C: n = 14). The biotechnical handling on infected mice are indicated below the axes (CS: Clinical scoring, B: Bleeding, †: euthanasia and organ harvesting). Gray bars indicate the phases of infection.
(TIF)

S2 Fig Distinctive viral antigen associated gene expression. Heatmaps showing KC and IM specific genes in other cell population, addition to Fig 5.
(TIF)

S1 Table Relative RNA counts of sorted Kupffer cells and infiltrating monocytes. (XLSX)

## Abbreviations

Cl13: Clone 13
DMEM: Dulbecco’s modified Eagles Medium
EMEM: Minimal essential Medium Eagle
FCS: Fetal calf serum
HBV: Hepatitis B virus
HCV: Hepatitis C virus
iNOS: inducible nitric oxide synthase
IM: inflammatory monocytes
IL: interleukin
IFN: interferon
ISG: interferon stimulated genes
*i.v.*: intra venous
KC: Kupffer cell
LCMV: Lymphocytic choriomeningitis virus
MDSC: myeloid derived suppressor cells
PFU: plaque forming units
TNF: Tumor necrosis factor
