# Supplementary figures and images for: Liver Monocytes and Kupffer Cells Remain Transcriptionally Distinct during Chronic Viral Infection
Source: PLoS One. 2016 Nov 3;11(11):e0166094. doi: 10.1371/journal.pone.0166094 (PMC5094584; doi:10.1371/journal.pone.0166094)

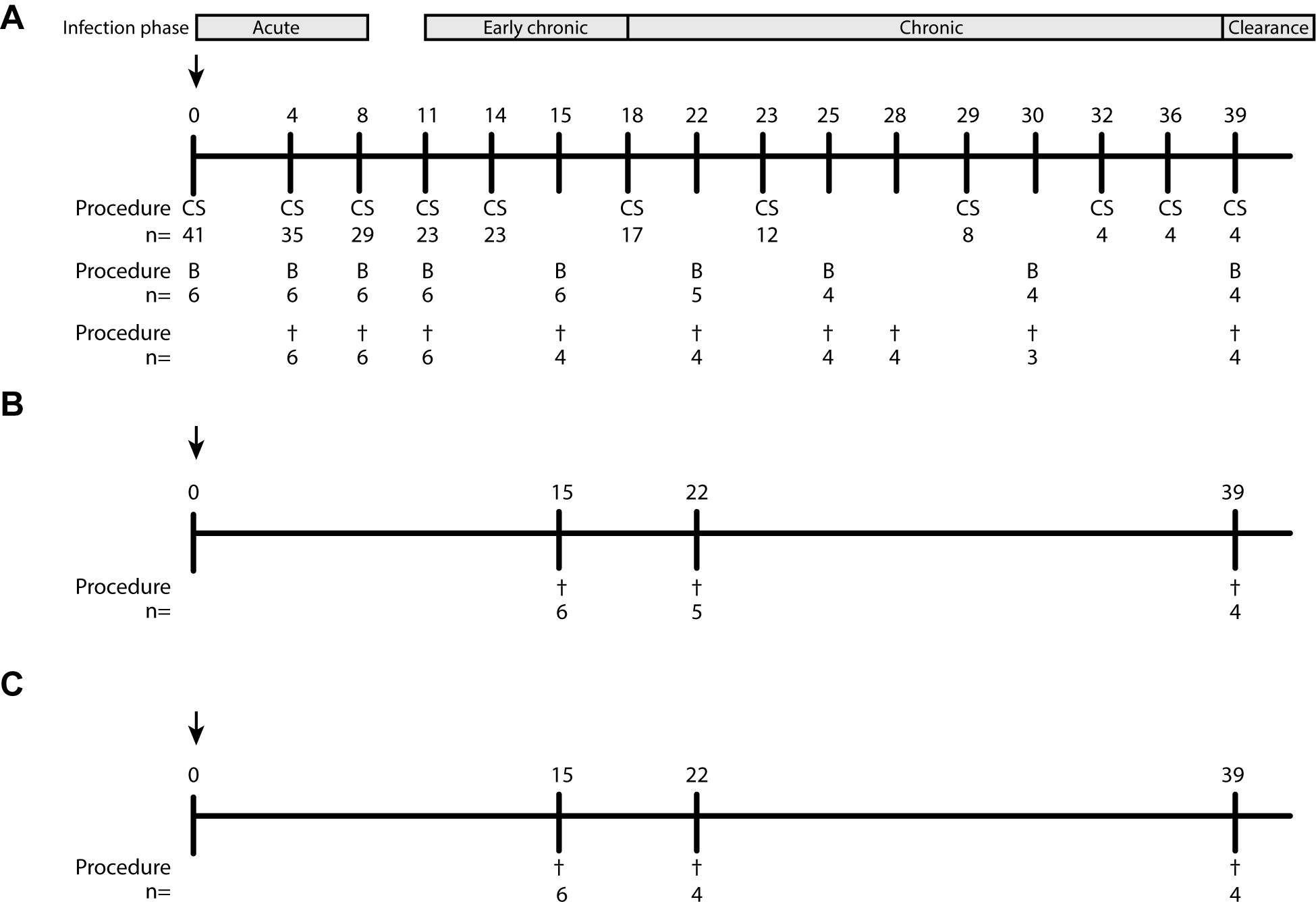

Supplement: S1 Fig — For each experiment uninfected control mice were sacrificed at day 0 (A: n = 6, B: n = 6, C: n = 12). A total of 70 mice were i.v. challenged after clinical scoring and weighing with 2x10E6 PFU LCMV Clone 13, indicated by an arrow (A: n = 41, B: n = 15, C: n = 14). The biotechnical handling on infected mice are indicated below the axes (CS: Clinical scoring, B: Bleeding, †: euthanasia and organ harvesting). Gray bars indicate the phases of infection. (TIF) [file pone.0166094.s001.tif]

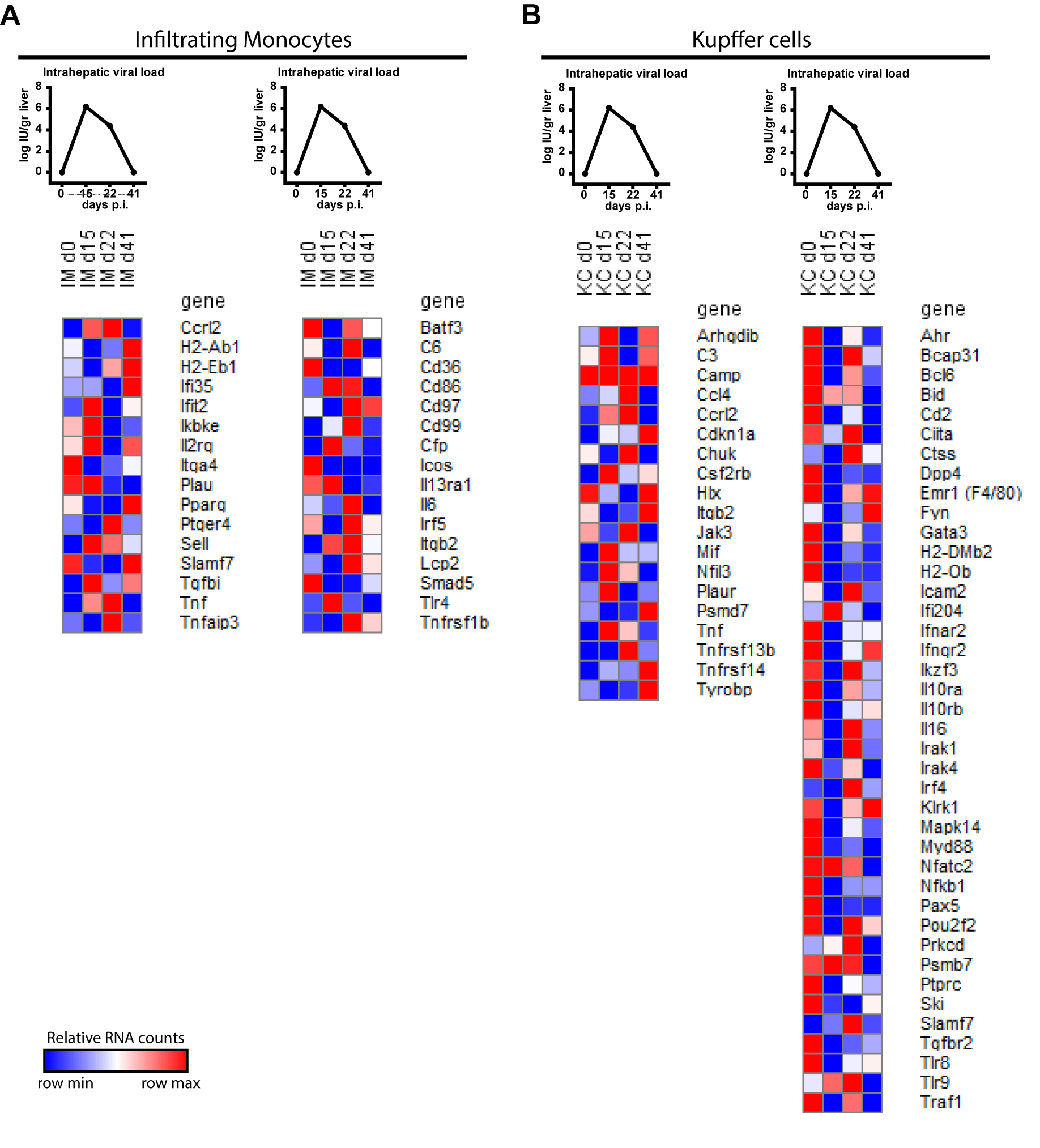

Supplement: S2 Fig — Heatmaps showing KC and IM specific genes in other cell population, addition to Fig 5. (TIF) [file pone.0166094.s002.tif]
